# Older women’s lived experience of ageism: Breast cancer screening beyond the targeted age range

**DOI:** 10.1177/17455057251400246

**Published:** 2025-12-24

**Authors:** Joanne Adams, Virginia Dickson-Swift, Irene Blackberry, Eva Yuen

**Affiliations:** 1 Violet Vines Marshman Centre for Rural Health Research, La Trobe Rural Health School, La Trobe University, Bendigo, VIC, Australia; 2 Care Economy Research Institute, La Trobe University, Wodonga, VIC, Australia; 3 John Richards Centre for Rural Ageing Research, La Trobe Rural Health School, La Trobe University, Wodonga, VIC, Australia; 4 Centre for Quality and Patient Safety Research; Institute for Health Transformation; Deakin University, Geelong, VIC, Australia; 5 School of Nursing and Midwifery, Faculty of Health, Deakin University, Burwood, VIC, Australia; 6 Monash Health, Clayton, VIC, Australia

**Keywords:** ageism, communication, older women, qualitative research, health services

## Abstract

**Background:**

Ageism has been described as a form of prejudice of one age group against another and is experienced in a range of ways. Little is known about the impact of ageism on the health and well-being of older women. Interacting within the healthcare system becomes more complex with age. This occurs because of increased comorbidity and changing perceptions of the ageing body.

**Objectives:**

This study aimed to explore the concept of ageism by reviewing older women’s perceptions and experiences of accessing breast cancer screening.

**Design:**

Drawing on data gathered by the same researchers, this study utilises a secondary qualitative analysis of primary data.

**Methods:**

This study draws on a purposive sample of 60 women aged ⩾75 years who participated in an Australia-wide study conducted in 2022. In-depth individual interviews were audio-recorded, transcribed verbatim and imported into NVivo software (Version 15) to enable thematic analysis. Secondary analysis drew on a theoretical lens of ageing together with researcher reflection of ageism in society.

**Results:**

Women in the study reported experiencing changes in their interaction with and perceptions of healthcare based on age. Perceptions of ageism were grouped into theoretical themes relating to micro, meso and macro concepts of ageism. Thematic headings included: ageing with self-determination and autonomy; meeting the changing health needs of older women and the invisible generation. Generational differences, health system experiences, societal influences and use of technology and online platforms were discussed. Results indicate that ageism may be based on both perception and experience. The impact on health and well-being of women aged ⩾75 years was variable and often modified by associated peer groups.

**Conclusion:**

Identifying and describing the multiple layers of ageism presents healthcare providers with an opportunity to understand how ageism is experienced by older women. This has implications for effective communication and care. Health messages designed to reach older women must be cognizant of both implicit and explicit forms of ageism. Establishing trust and avoiding reliance on online platforms as sources of information are important for this age group. Health promotion and prevention initiatives based on age-related criteria may contribute to notions of ageism.

## Introduction

The Global Report on Ageism launched in March 2021 by the World Health Organization (WHO), High Commissioner for Human Rights^1^ highlights the impacts of ageism on health and well-being outcomes for older people across the globe. While ageism as a concept has received much attention in the literature over the past 50 years, ageist attitudes and policies continue to be developed and implemented around the globe.^2^ Combating ageism continues to be a key area of work internationally and is one of the four key action areas of the United Nations Decade of Healthy Ageing (2021–2030).^2^

The WHO defines ageism as referring to situations in which people are treated with discrimination, prejudice and stereotypes because of their age.^3^ Ageism is highlighted as an ‘invisible phenomenon’, particularly because it is initiated by how we think, feel and act towards people based on their age.^4^ Ageism can include negative attitudes and feelings about the ageing process, old age and older people and can lead to discrimination.^5^ Elements of ageism can be found in individuals’ behaviour, in organisational regulations and in cultural values.^6^ Ageism has been compared to other types of discrimination including sexism and racism.^7,8^ Butler highlighted how ageism manifested as attitudes, behaviours and institutional practices and policies directed towards older adults and describes impacts to both physical and mental health resulting from ageism.^8^

The effect of both structural and individual level ageism on older people across multiple health domains has been demonstrated in a recent systematic review undertaken by Chang et al.^9^ The review included 422 studies from across the globe and concluded that ageism led to significantly poorer health outcomes in 95.5% of the included studies. The results of the review highlighted that ageism negatively influences the health of older people through psychological, behavioural and physiological pathways.^9^ This included the impact of negative self-perception relating to work opportunities and capacity, propensity for physical activity and presence of stress biomarkers related to inflammation. A significant adverse relationship between ageism and health is predicted to get considerably worse over time. This underscores ageism as a social determinant of health with implications similar to those associated with racism.^9^

Despite being the highest consumers of health interventions and medications, discrimination encountered by older people on the basis of age is experienced in a range of ways.^10^ Notably, the systematic exclusion of older people from randomised clinical trials within pharmacological, behavioural and clinical contexts. This has been described as a form of ageism in a number of systematic reviews.^10–12^ Reasons given for exclusion typically focus on the additional time and costs incurred in enrolling older people. This gives rise to the suggestion that older people are viewed as an economic burden and a drain on scarce resources.^13^ Much of the available evidence comes from trials with younger people.^14^ Bumanlag et al. describe the use of drugs for cancer patients that are not necessarily well tolerated by older people.^14^ This has clear and important implications for health professionals required to advise older patients of the best course of treatment/action without clear evidence supporting its effectiveness for older people.^13,14^

While ageism affects both older men and women, older women are more likely to be targets of ageism^15^ often experiencing what has been termed a ‘double jeopardy’ due to the combined effects of ageism and sexism.^16^ Stereotypes related to age can compound the issues created by sexism which can increase the potential for devaluation of women’s health concerns.^17^ Self-devaluation can deter some women from seeking necessary care affecting access to proper treatment.^18^ Merodio et al. further describe gendered ageism as intersectional, contributing to invisibility and homogenisation of older women, such that it may harm their health and quality of life as well as limit social participation.^18^

Attitudes and behaviours related to age can manifest in a number of ways including rationing care, patronising behaviours and self-limitation in response to internalised devaluing of self.^1^ It can be demonstrated through the explicit use of age cut-offs for treatment or resource allocation or through implicit age-related biases which seek to limit access or create barriers to healthcare.^13,19^ Implicit and explicit ageism can lead to inadequate or inappropriate care, decreased or delayed access to healthcare services, resulting in decreased survival, poorer quality of life, increased cognitive and functional impairment and increased medication non-compliance, emergency visits and hospitalisations.^9^

Recognising the multiple layers of ageism provides healthcare providers with an opportunity to understand the impact and how this may be mitigated in forms of communication and care. Ayalon and Tesch-Römer^6^ acknowledged that ageism operates across three levels-micro, meso and macro. At the micro level, ageism relates to the individual (thoughts, emotions, actions), at the meso-level, it relates to groups, organisations and other social entities (e.g. in the domain of work or healthcare services); and at the macro-level, it relates to cultural or societal values as a whole (e.g. political regulations).^6^ Within the healthcare setting, ageism is particularly concerning for older women who are frequent users of the healthcare system both for their own health concerns and for the health concerns of others due to their caring roles.^20,21^ Ageist beliefs and stereotypes can interfere with older women’s own healthcare seeking, diagnosis and treatment recommendations and can contribute to gender disparities if they are perceived as too frail to undergo aggressive treatments.^20^

Access to all types of healthcare from preventive screenings (e.g. mammography) to expensive life-sustaining treatments are limited by policies and attitudes related to age.^13,19^ The biggest risk factor for the development of breast cancer in women is age; however, when age-related decisions and subsequent age limitations are imposed for access to screening services, this can reflect ageism.^19^ Internationally, there are a range of guidelines for breast cancer screening for older women, and national screening programmes are active in many high-income countries including Australia, Canada, Denmark, England, France, Ireland, Japan, South Korea, New Zealand, Sweden and the Netherlands. The benefits of continued screening for older women are controversial^22–24^ with uncertainty around benefits for older women attributed to a failure to include women over 74 years in randomised trials of screening mammography.^25^ As a result, the U.S. Preventive Services Task Force guidelines warn there is insufficient evidence to balance the benefits and potential harms of screening in this population group^26^ specifically, premature treatment for women likely to die with, rather than from, breast cancer.

Recommendations for breast cancer screening for older women focus more on an individualised approach taking into account potential benefits and harms in the context of a woman’s overall health status, life expectancy and prior screening history.^26,27^ A recent summary of recommendations from 21 high-income countries with the highest per capita spend on healthcare reported the most common screening age ranges as 50–69 years, although some countries recommend screening up to 74 years (e.g. Australia, Japan, France, Canada, Sweden and Netherlands) with a 2-year screening interval.^28^

Little is known about the impact of direct and indirect ageism on the health and well-being of older women particularly regarding health preventive behaviours like breast cancer screening via mammography. Drawing on a primary study published by the same authors^29^ consisting of qualitative interviews with Australian women ⩾75 years, this secondary analysis aimed to explore the concept of ageism by reviewing older women’s perceptions and experiences of accessing breast cancer screening.

## Methods

### Design

Qualitative secondary analysis (QSA) is a useful method to use for research when researchers wish to (1) investigate questions that are different from the primary study,^29^ (2) apply a unique theoretical perspective and/or (3) extend the primary work.^30,31^ The QSA undertaken for this study aimed to address all of these.

The primary study was granted approval by the La Trobe University Ethics Committee, Approval Number 21249.

### Participants and recruitment

A purposive sample of 60 women aged ⩾75 years was recruited from the general population for the primary study.^32^ Recruitment followed a range of sampling methods shown to be successful in recruiting older people.^33^ A range of print and social media channels within regional and national platforms were accessed. This included health service providers, consumer organisations, advocacy networks and community-based organisations who regularly engaged with older women. BreastScreen Australia within respective States also assisted with recruitment, promoting the study through internal communications. Network/snowball sampling (including word of mouth between older women themselves) was an important feature of recruitment. An overview of the participants is provided in Table 1.

**Table 1.** Participant characteristics.

Sociodemographic characteristics (*n* = 60)	*n*	%
Mean age: 78 years (SD = 3.9, range: 75–86 years)		
Geographic location		
Metropolitan	36	60.0
Regional (large town)	10	16.7
Rural (small town/remote community)	14	23.3
Country of birth		
Australia	41	68.3
Europe/UK	7	11.7
America’s	2	3.3
Asia/Pacific	6	10.0
Middle East	3	5.0
Africa	1	1.7
Education		
Primary school/some of high school	24	40.0
Year 12	2	3.3
Bachelor’s degree	19	31.7
Master’s degree or higher	5	8.3
Tertiary (international)	7	11.7
Unknown	3	5.0
Living situation		
Single (1)	25	41.7
Single (2+)	5	8.3
Married (2)	27	45.0
Married (2+)	3	5.0

SD: standard deviation.

### Data collection

Data from the primary study was collected via individual semi-structured interviews utilising an interview guide. Interviews were conducted by telephone or online video platform (i.e. Zoom) by JA, VDS and EY. An extensive scoping review of the current literature and review of government reports regarding breast cancer screening in Australia informed the interview guide.^34^ Interviews were digitally recorded with participant consent and transcribed verbatim.

The interview guide from the primary study was utilised as an iterative tool. While questions relating to ageism were not specifically included in the guide, a clear pattern of discussion relating to ageism organically emerged. This was subsequently woven into ongoing interviews.

### Data analysis

Within the primary study transcripts were independently reviewed by two researchers (JA and VDS) and an initial thematic coding framework using Braun and Clarke’s^35^ six-step process was developed. Interview transcripts were imported into Nvivo^36^ software to support analysis. The Consolidated Criteria for Reporting Qualitative Research (COREQ)^37^ was utilised to ensure methodological rigour and transparent reporting (see Supplemental Material). The research team met regularly throughout the analysis process to share coding and to confirm theme development and refinement.

For this secondary analysis, we have chosen to view primary data through a theoretical lens described by Ayalon et al.^6,38,39^ in relation to ageism. This places the aetiology of ageism within three interrelated levels: micro, meso and macro. Our analysis of primary data acknowledges these levels together with a reflexive approach acknowledging the deeply personal impact of perceived ageism.^40^ Researcher reflection provided an important basis for discussion of ageism during interviews. It transpired that researchers conducting the interviews each had mothers who fell into the age category being discussed. This provided some insight to the lived experience of ageism experienced by women over 75.

In this context, themes are contextualised as patterns of shared meaning united by the aetiology of ageism. This describes a micro level of ageism that is characterised by an awareness of generational differences and concepts of self. At the meso level, women described experiences in relation to the health system, societal interactions/influences and use of technology and online platforms. At the macro level, women described an awareness of the impact of their age, and how this influenced societal perceptions and interactions.

**Figure 1.**
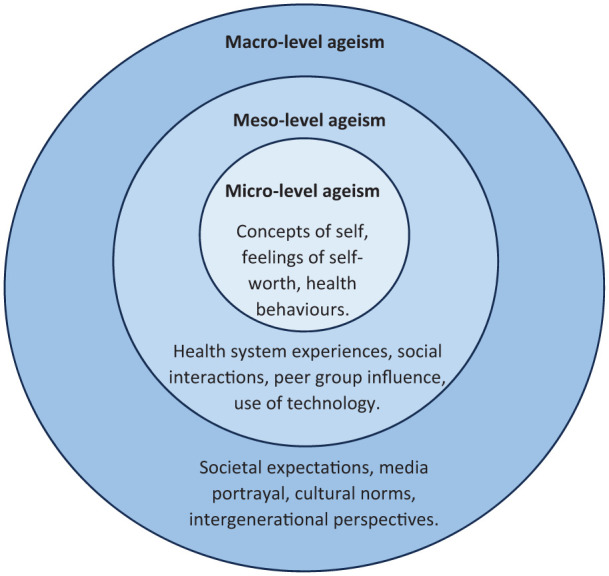
Diagrammatic representation of study themes.

Analysis has been conceptualised using the following diagram. The diagram depicts the levels of ageism described by Ayalon et al. and includes some of the main topics drawn from our data. The diagram visually demonstrates the compounding impact of each level of ageism, with concepts of self as a central core progressing through layers of experience to the broader views of society. Individuals may experience each layer of ageism differently with some describing awareness of all three and others focusing more on one or two layers. Such experiences may also change over time and with different circumstances (Figure 1).

## Results

Within the primary study 60 women aged ⩾75 years volunteered to participate in an individual interview. A range of socio-demographic information was gathered (see Table 1). The age range of the participants was 75–90 years with a mean age of 78.4 years. Of the 60 participants, 47 (78%) were regular breast cancer screeners, 10 (17%) were non-regular breast cancer screeners and 3 (5%) had never undergone breast cancer screening.

The following key themes regarding ageism were developed from the analysis of transcripts. Key quotes are presented verbatim *in italics* throughout.

### Ageing with self-determination and autonomy (micro-level ageism)

The perception women held of themselves with regard to their age was influenced by a range of factors. This included reference to their existing (and past) health behaviours, expectations and experiences. A desire to maintain appropriate levels of independence was often expressed. This was consistently framed by how well equipped they felt to achieve desired outcomes.

Compliance to the instructions of doctors and authority figures was a common feature of women in this age group, particularly for those without any medical/nursing training. For some this softened with time and experience, but for others it remained a feature of the doctor–patient relationship and influenced the nature of conversations with health professionals.

*Because a lot of women, you know, my age, . . . wouldn’t dream of asking a question. . . . And they might violently disagree . . .No, a lot of older people kowtow*. P.10, Age 80.

*Yes, and I think at our age, we’ve come through a lifetime of being compliant people because we never questioned people during the Second World War. . . . our parents didn’t have much, mostly. So we just accept life, that this is the route that life has forward. . . . we stand might stand up and argue the point. But basically, I think loads of women I know are compliant, intelligent women*. P.11, Age 78.

*. . . What the teacher said, what the priest said, what the doctor said, it was that respect for authority. And also a lot of people of my generation or even and older, didn’t, didn’t have a high level of education . . . And they were aware that these people in authority were learned people*. P.41, Age 80.

They were aware that younger women did not feel obligated to follow the instructions of health professionals and questioned directions more readily.

*The young ones say, oh lets, you know, we don’t have to do that. You know they’re different all together really*. P.22, Age 78.

Health behaviours of older women were also influenced by an awareness that previous generations of women did not have access to screening. Knowledge of breast cancer within their previous generation was minimal and outcomes from treatment were often poor.

*No, there wasn’t any, any breast cancer around at all. This is why it was sort of, you know, quite a surprise, although going back years ago, half of them wouldn’t have even known that they had it probably*. P.35 Age 87.

Older women were often highly vigilant with screening because they had witnessed family members experience breast cancer at advanced stages (without the benefit of screening).

*I think because I’ve been surrounded [by people diagnosed with cancer]. Quite a few in-laws have had breast cancer and my mother-in-law. . . . she found out she had it when she was in her 80’s, . . . she was about 84, I think when they found it and like her daughter was quite shocked because . . . I don’t know whether she had an abscess or something that was showing on the outside. . . . it was something you could tell that wasn’t right. . .just from looking at it*. P.18, Age 81.

A strong desire to remain independent was evident amongst many participants. Self-determination and autonomy were valued despite a tendency to compliance. This extended beyond vigilance regarding their health and included continuing to drive a car, maintaining their own house and garden.

*. . . I’ve got family from one side of . . .capital city. . . to the other and I’m on the road constantly driving quite. . .long distances, three quarters an hour to my sons homes. . .. You know you’ve got to keep up with it. . ..You’ve got to get out there amongst it*. P.25, Age 86.

*[Other people] have all these help, these women coming in and doing the housework. . . Well, I think. . .if you don’t use it, you lose it. And I think that’s a lot to do with it, too. With the older ones, you know, relying on other people, you’ve got to sort of be a little bit keep yourself a little bit independent and do as much as you can yourself too*. P.35, Age 87.

Some women felt that their experience of age was not the same as previous generations because they were able to stay active and involved in a range of activities. This was highly valued.

*And these days, 75 is not how can I put it? It’s not as old as perhaps it was once. . . . Like all the 75 year olds that I know, are well and truly involved in all sorts of, you know, things in society*. P.44, Age 75.

*You have a lot on your mind. I don’t know about others, but, you know, I’m still I’m involved in several committees for breast cancer. I’m also involved in a group that looks after refugees. I’ve got, you know, grandchildren that I’m responsible for. So, you know, it’s still a very busy life*. P.44, Age 75.

Keeping busy and helping other people gave a strong sense of purpose and identity for another participant.

*I’d go crazy if I didn’t have anything to do. . .This morning I went and did two ladies shopping here, plus my own, and so that filled my morning in. Then I’ve come home and I’ve put soup on and I’ve put a piece of corned beef on. . . Yesterday I spent all day at the kiosk, and then the afternoon I spend it at the dementia section*. P.46, Age 79.

### Meeting the changing health needs of older women (meso-level ageism)

Participants discussed experiences within the broader health system related to their age. For this generation of women, ageism can be seen to combine with sexism (discrimination based on gender) in unique ways. This is because a strong (often gendered) sense of compliance to authority figures is coupled with a felt sense that their changing needs are not recognised by the health system. These distinct and compounded forms of discrimination could be seen as intersectional in nature. Older women were aware of the large disparity in health status within their age group and expressed concern that generalisations were made by health professionals that influenced their care.

Some participants felt that health professionals did not communicate well with older people and could not relate to the difficulties they faced.

*I think we’re ageing, but we’re living longer. And we’re living longer with some forms of disability. . . . But you feel like you’re swimming upstream a bit. . . . I think [providers] really need to relate to the ageing person more*. P.02, Age 76.

*You know, the people, decision makers, they’ve got a limited experience of how things are in life. Really, and so, . . . as that demographic gets younger, it gets worse for us. Because you just get pushed just off the edge of it, off their paper. . . . We just don’t figure into any of their calculations*. P.21, Age 75.

Other women felt that the health system treated older people differently in terms of the care they received. Older women discussed this amongst themselves but had limited opportunities to discuss perceived inequities with health professionals.

*Once you reach 75, it’s almost a cut off point in most fields. . .the discussion I have with women of my age is that it is that you reach that age and congratulations. And now we’ll just keep you alive, but we won’t be specific in the care of you*. P.11, Age 78.

*I’ve had many different kinds of things, especially . . . after my 80th birthday . . . which resulted in in a fair amount of spasticity. And people, doctors would say, Well, you are 80, you know, . . . And I said, . . . before this happened, I was walking three kilometres before work every day. I was important in my work. I was getting really good results in my work. I can’t do that now. And just because I’m 80 doesn’t mean we stop trying to fix things*. P.13, Age 82.

Technology was also a concern for participants as it had become the means of obtaining health-related information. For some this created a form of social inequality that was difficult to overcome. The use of technology increasingly influenced their capacity to continue interacting in the world beyond their own home.

Some women had minimal access to digital devices and had limited understanding of how to use them correctly. This was highlighted for one participant when COVID tracing via mobile phone was prevalent.

*I don’t have a mobile phone because the people I know that have got them, they can never they can’t do. . .the Covid thing. I’ve got mine on paper. . .they got the phone and you tried to ring them, and nothing. And I think, oh well, I will have to get one eventually, I know. But for the time being, no*. P.20, Age 78.

*I’ve got a laptop. And that’s the extent of my involvement, um electronically. . . . I can find out what I want to more quickly by talking rather than typing*. P.28, Age 77.

There was a strong awareness that information regarding health was prominently available from online sources; however, lack of access to and knowledge of how to use digital devices were barriers to accessing online information for older women.

*. . . it’s potentially very isolating because. . .younger people are relying on people accessing information from electronic sources. And if that’s not happening [for older people], then you’re not getting the information*. P.25 Age 86.

*. . . when they say, you know, go to dot com, dot au, you know, dot, dot, and you think, well, . . . how can I do that because I haven’t got a computer. And you’ll find that a lot of the older ones at this stage would not have a computer. . . So I think this is where it’s falling down. And what how do you fix that? I don’t know. Unless you send us all back to school again*. P.35 Age, 87.

Others resisted using technology and online activities because it gave them a reason to leave the house and remain active in the community. This was valued as a means to remain healthy, alert and independent. The use of technology held limited interest for many older women beyond the need for communication.

*. . . I probably could learn it if I wanted to. But the other thing I, I feel like when I get my gas bill, well, I have to go into town and pay that. So that makes me do something. . .and my daughter keeps saying to me, Mum, look get on the computer and do your banking. And I said, No, no, no, I’m driving. Maybe it might come that way. . . But at the moment I can drive and that gives me something to do and they’re just sort of slowly cutting everything off for our age grouping, if you know what I mean*. P.46, Age 79.

### The invisible generation (macro-level ageism)

While breast cancer screening provided a valuable example of ageism within the healthcare system, participants described societal influences from a range of interactions within the broader community with regard to age. Some recounted what they saw as commonly accepted perspectives of younger generations towards older people and how these influenced actions and behaviours.

In considering the change in notification regarding access to breast cancer screening services, participants expressed a view that perhaps they lacked societal value. Accessing services clearly targeted at younger generations triggered thoughts that they might be seen as a burden on younger generations. This was noted in how they interacted with younger generations and the busy lives of younger people, including those in the medical profession.

*I suppose it is really that we lose value as the age increases. And I see it often. And I feel sorry for young people in a hurry if I’m in the way. And mostly when I apologise, they smile and say it’s fine. But sometimes you get that irritation. And that’s bound to be. They’ve got their lives to live and. . .I do get in the way with my walking frame all the time. . .So I guess it’s there. And if it’s there in the young people that we meet, it’s certainly there in the medical profession as well. They’re not known to be in front of the rest of society in things like that*. P.13, Age 82.

*Yes, and I think one other thing I’ve noticed is, you know, when you’re going through the checkout in the supermarket. . .for instance [and you have a] card. . .you haven’t got the electronics thing. . . if there’s a line you can sort of feel people [thinking] ‘Oh, hurry up, that old person, that old woman in front of me is taking so long’*. P.17, Age 76

Some participants felt that they became almost invisible within society and that their needs were not considered important and were largely misunderstood.

*Um, well, it’s a bit “typical”, you know, because they don’t, generally speaking. I think, they think that women especially over 75 don’t exist. . . . There’s so few of us*. P.21. Age 75.

A sense of invisibility, however, was also shared with a sense of comradery amongst peers. Older women empathised with one another and offered support to overcome negative feelings associated with perceived ageism.

*The invisible people. My sister always says, you know, she’s three and a half years younger than I am. But she said, oh, it’s awful. . .You’re just an invisible old person. And I said, Well, yes, but we are just invisible old people to the rest of the world now. But that doesn’t matter. We’re ourselves. We’ve just got to make the best of our lives*. P.25, Age 86.

Others relayed how members of their own family treated them differently because of their age. This in turn influenced the self-perception of women (in relation to age) in both negative and positive ways.

*Your children treat you differently. One of my sons treats me like a small child, and I love him for it. But people do treat you differently and that, it makes you think, Oh gosh, I really am getting old now*. P.13, Age 82.

*I mean, I think I’m fortunate in that I’ve got grown up kids who sort of encourage the role of grandparent in the family, that it’s a role that is valued. And the. . . encouragement to keep having adventures. . . I’m fortunate in that. . .[otherwise] it would be easy to feel that you’re being discriminated against because you’re old*. P.50, Age 83.

## Discussion

This secondary analysis represents one of very few explorations of the lived experience of ageism in Australia. It clearly relates the challenge of accessing health services as an older woman and maintaining an aptitude to continue interacting more broadly within society. The experiences of women were characterised by perceptions of ageism that could be seen as intersectional in nature. Findings reflect often very personal accounts of the older ‘self’ in relation to the younger ‘self’ and to those they interact with. Such perceptions were heavily influenced by societal norms, healthcare systems, infrastructure and methods of communication that enable (or disable) active participation.

Our analysis demonstrates that ageism was experienced on multiple levels which resulted in a compounding effect. This was influenced by varying levels of self-perception and interaction within the healthcare system, broader society and friendship and family groups. Within the healthcare setting, ageism is particularly concerning for older women who are frequent users of the healthcare system both for their own health and for the health of those to whom they provide care.^20,21^ Beliefs and stereotypes related to older age have the potential to interfere with older women’s own healthcare seeking, diagnosis and adherence to treatment recommendations and can contribute to gender disparities if they are perceived as too frail to undergo aggressive treatments.^20^

The notion that self-perceived ageism impacts health and well-being of older women is supported by other studies that describe indirect consequences for ongoing well-being.^41,42^ This highlights the importance of the element of choice and informed decision-making.^29^ Facilitating informed decision-making for this age group appears to address not only the basis of self-perceived ageism but also informs the actions of health providers (who may unintentionally perpetuate ageist practices). Results from a recent study that aimed to support informed decision-making of older women with regard to breast cancer screening indicated that providing a clear rationale for an upper age limit was beneficial. Increased levels of informed decision-making led to a reduced desire of some older women to continue screening.^43,44^

It remains, however, that such conversations are complex and must account for many individual and systemic variables.^43,45–47^ Health professionals may have limited knowledge of the benefits and harms of ongoing screening.^48^ Evidence continues to suggest contentious points of view regarding the benefits^49,50^ and harms of screening women after the age of 75.^43,51^ Health professionals may require more support to further explain this information to older women.^52–54^

Individual ageism includes the impact of culture-based negative age stereotypes and negative self-perceptions of ageing on the health of older persons.^9^ Within our analysis, this was reflected in an awareness of being treated differently, being ‘invisible’ and losing value. These negative self-perceptions of ageing can, in turn, impact on physical and mental health^55^ and play a role in the development of social isolation, depressive symptoms and withdrawal.^56,57^ Previous studies have shown that older people with negative self-perceptions of ageing were significantly more likely to have functional decline than those who held positive self-perceptions.^58–60^ Yet negative self-perceptions are also drawn from repeated exposure to systemic forms of ageism. The language of ageism is highly complex and difficult to identify, however, because it is rooted in both explicit actions and implicit attitudes.^61^

Structural ageism is used to explain the explicit or implicit policies, practices or procedures of institutions (e.g. hospitals, healthcare, education) that discriminate against older people and reflects both meso and macro levels of ageism. Many women in our study felt that moderating access to breast screening based on a target age range represented just one amongst other forms of ageism. No longer receiving notification or a reminder to attend screening after the age of 74 was significant to many women who described this amongst other experiences of being treated differently as an older person within the healthcare system. Structural ageism can also include ‘age-based’ actions of individuals who are part of these institutions (including doctors and nurses).^62^ This was evidenced by one participant who felt that younger doctors and nurses entering the health system have increasingly less understanding of the needs and concerns of older people.

Many participants in this study were regular breast cancer screeners prior to reaching the age of 75. They typically held proactive views towards their health in their younger years. It could be argued that women who are proactive in managing their health do not typically have negative self-perceptions. With age they remained proactive and increasingly valued autonomy and independence. In some respects, this runs counter to the above studies which imply that older people who hold negative self-perceptions and are more likely to infer ageism from systemic sources. This might suggest that ageism is experienced more broadly than previous studies propose. It also raises the question of whether or not younger people can recognise unintentional ageism without the insight of older people.

Very little is actually known about the risks and benefits of breast cancer screening for this age group.^29,34^ While Chang et al.^9^ point out the structural ageism that emerges from excluding older people from clinical trials, with limited evidence to support or refute an exclusion rationale, it is clear that this leaves us with an apparent ‘stalemate’. Actively discouraging older people from screening, means that sufficient data to explore the harms and benefits of screening does not exist. Those tasked with explaining to older women the benefits and harms of screening have a limited evidence base from which to draw. Older women are inevitably left to make a decision based on their own experience and that of their peers.^63^ This may inadvertently reinforce structural ageism.

Structural ageism (at both meso and macro levels) may also be evidenced by a growing reliance on technology as the primary means of communicating information regarding health and healthcare. Often this is intended to replace and/or supplement face-to-face conversations. Online booking is also increasingly used to arrange appointments with health professionals with women describing their frustration regarding access to technology and expectations for its use. Using online platforms, for some, led to fears of reduced interaction within broader society and took valued activities away from their regular routines.

Our analysis demonstrated the importance of personal experience together with peer group conversations amongst older women. This was consistent with other studies exploring the decision-making of older women regarding breast cancer screening specifically and health more generally.^64,65^ Older women are generally compliant to the authority of health professionals,^34^ yet women reported that appropriate conversations with health professionals were not occurring. If they did occur it was generally to emphasise the benefits of screening rather than the harms.^65,66^ The capacity of health professionals to relate appropriately to older people may be a factor in this. So too is the awareness many women held that the health status of their peers aged ⩾75 years was highly variable and uniform health advice was not necessarily trusted or helpful.^66^ Quality of life, independence and personal autonomy were important features of women’s decision-making regarding their health and breast cancer screening represented just one more way of maintaining this.

### Strengths and limitations

This secondary analysis provided a great opportunity to bring to light concerns expressed by our participants beyond the initial research questions of the primary study. It was clear there was another story to tell regarding access to healthcare generally and preventive healthcare specifically for older women. This was born out through in-depth interviews that were semi-structured in nature. The secondary analysis was conducted by the same authors as the primary study. This enabled consistency in interpreting results and allowed full access to primary data.

The largest group of participants in the study were regular breast cancer screeners prior to reaching age 75. Many clearly intended to continue screening into their later years. This may mean they were more inclined to express concern regarding perceived barriers to ongoing screening and more attentive to the implications of age-based targeted screening. Similarly to the findings of Weller et al., regular screeners were more likely to be proactive in preventive health measures, actively seek personal autonomy and independence and generally experienced good health.^65^ This may conversely set them apart from participants identified as having negative self-perceptions and functional decline in other studies describing ageism within healthcare settings.^58–60^

## Conclusion

The lived experience of women accessing breast cancer screening services beyond the targeted age range suggests that existing healthcare services may not be meeting the needs of older women. Women aged ⩾75 years increasingly experience broad ranging health status. The use of target age ranges to guide preventive healthcare pre-supposes the health status of healthcare consumers and represents an implicit form of ageism. With limited evidence to support the rationale for age-based recommendations, healthcare professionals have limited capacity to give appropriate guidance to consumers. Adopting a strengths-based approach that enables shared decision-making and considers capacity and comorbidity will allow health professionals to avoid notions of ageism. Further measures may include sensitivity training for health providers to understand the needs of older adults. Importantly this may provide guidance to health professionals through the use of evidence-based decision-making guides. Additionally, ensuring advances in technology are balanced with pen and paper methods for those with limited access to digital devices would be beneficial for this age group. The ongoing contribution that older people make to society generally should not be underestimated. They not only provide an important source of guidance and role modelling for younger generations, but also the greatest source of comfort and reassurance to their peers who may experience worse health outcomes.

## Supplemental Material

sj-docx-1-whe-10.1177_17455057251400246 – Supplemental material for Older women’s lived experience of ageism: Breast cancer screening beyond the targeted age range Supplemental material, sj-docx-1-whe-10.1177_17455057251400246 for Older women’s lived experience of ageism: Breast cancer screening beyond the targeted age range by Joanne Adams, Virginia Dickson-Swift, Irene Blackberry and Eva Yuen in Women's Health
